# Telephone Consultation and Prescription in Pediatrics: Contributing Factors and Impact on Clinical Outcomes

**DOI:** 10.3389/fped.2019.00515

**Published:** 2020-01-15

**Authors:** Raymond N. Haddad, Celine Sakr, Lydia Khabbaz, Hayat Azouri, Bassam Eid

**Affiliations:** ^1^Department of Pediatrics, Hotel Dieu de France University Medical Center, Saint Joseph University, Beirut, Lebanon; ^2^Faculty of Pharmacy, Saint-Joseph University, Medical Sciences Campus, Beirut, Lebanon; ^3^Division of Pediatric Gastroenterology and Nutrition, Department of Pediatrics, Hotel Dieu de France University Medical Center, Saint Joseph University, Beirut, Lebanon

**Keywords:** telephone, mobile phone, consultation, drug prescription, pediatrics

## Abstract

**Objectives:** To evaluate phone-based consultation practices and drug prescription profiles in pediatrics and to highlight their possible uses, contributing factors, and effects on clinical outcomes.

**Background:** The ownership and everyday use of cell phones are increasing worldwide. Telehealth is gaining the support of health professionals for the delivery of simple healthcare measures to more complex management decisions. Despite this, in our country, doctors have been advised by concerned authorities to avoid any phone-based medical activity as the safety of such practices is still not well-established, especially among vulnerable pediatric patients.

**Patients and Methods:** This cross-sectional study was conducted on a national level over 5 months. Phone consultations and prescription behaviors data were collected through a self-administrated questionnaire. The target population consisted of pediatric-trained physicians with at least 1 year of experience. Factors influencing telephone prescriptions were assessed using bivariate analysis.

**Results:** Of among 120 included physicians (75.0% male), 64.2% were general pediatricians, 77.5% practiced in private clinics, and 27.5% had more than 20 years of work experience. All participants gave medical advice over the phone; 61.7% considered that they should be reimbursed for these activities and 29.2% of them reviewed 50% of their patients for the same complaint. A total of 109 participants (90.8%) prescribed drugs using a direct phone call (80.7%), SMS (27.5%), or WhatsApp application (61.5%). Antipyretics (97.2%) and cough suppressants (48.1%) were the most frequently prescribed drugs. Pharmacists' corrective interventions were seen in 40.4% of prescriptions. Fever was the only symptom that was statistically associated with phone prescriptions. Prescribers seemed to be less experienced and were more likely to consider phone-based practices as reimbursable activities.

**Conclusions:** Consultations and prescriptions through mobile phones are extremely frequent in pediatric practices, even when restricted by responsible authorities. Our results highlight the frequency of medical prescription errors and the need for corrective interventions by pharmacists. The current practice of telemedicine may not ensure the patient's safety but exists rather as a convenience. There is a need for proper oversight with a regulatory framework and input from all stakeholders, including pediatricians and pharmacists.

## Introduction

The healthcare sector is experiencing continuous improvements due to the implementation of innovative technologies, especially in the field of telemedicine. It offers new modalities of medical information exchange, more efficient communication, reduced costs, improved adherence to treatment, and better clinical outcomes (1, 2). With this in mind, mobile phones and related applications seem to be promising tools in medicine and are often used by patients worldwide to benefit from free medical advice quickly and easily (3, 4). In addition, recent access to voice and video real-time communication services allow clinicians to work more effectively with their patients, especially in pediatrics (5). As most people in our country have ready access to cellphones, physicians have provided phone-based medical consultations. Regulatory bodies have advised against this practice, as patient care and safety could be compromised. The role of telehealth in medical practice remains highly controversial for many clinicians as telephone consultations and prescriptions are not risk-free and may result in miscommunication and harmful medication errors (6). In pediatric care, parents are generally responsible for their child's health rather than the patients themselves. A pediatric medical check-up involves many specific challenges, requires special skills, and should be always based on a triad: the pediatrician, the child, and the caregiver (7). Given the unique characteristics and vulnerability of the pediatric population, healthcare professionals need to evaluate the child and to deal with their caregivers. We hypothesize that anxious parents may consult pediatricians more readily on the phone for every concern about their child, leading to higher rates of telephone prescriptions. For all of these reasons, and for the first time in the Arab world, we decided to conduct this study to evaluate this practice among pediatric-trained physicians and to highlight its possible uses, contributing factors, and likely effects on clinical outcomes.

## Patients and Methods

### Study Population and Design

This is a descriptive, observational, and cross-sectional study that was conducted over 5 months (between January 2019 and May 2019). During the study period, data were collected from all over the country through a self-administrated questionnaire that was distributed by our assistants to Lebanese pediatric-trained physicians with at least 1 year of experience at Saint Joseph university teaching hospital, Hotel Dieu de France, in private pediatric clinics and mainly during regional pediatric scientific meetings that were held in different regions of the country. The questionnaire was written in English to be understood by the majority of the participants. It included 21 questions and evaluated major information about the physicians' specialty and relevant data related to telephone consultations and prescriptions without disclosing any personal identifiers. Anonymized questionnaires were collected by our assistants and vetted by the authors of the study.

### Statistical Analysis

Statistical analyses were performed using the Statistical Package for the Social Sciences Statistics (SPSS), version 22 for Macintosh (IBM, Armonk, NY). Categorical variables were reported as frequency and percentage. Statistical analysis was conducted using the Chi-square test and Fisher's exact test as appropriate. A *P* < 0.05 was considered indicative of statistical significance. All reported *P*-values were two-sided.

## Results (Tables 1, 2)

The rate of participation in this study was 96.0%. Five pediatricians did not fill the questionnaire due to a lack of time. A total of 120 physicians were included in this study; 75.0% were men and 64.2% were general pediatricians. Among pediatric specialists, 37.2% were neonatologists and 16.3% were pulmonologists. The majority of the pediatricians (77.5%) practiced in private clinics and teaching hospitals (49.2%), while only 17.5% worked in governmental health facilities. Over one quarter of the respondents (27.5%) reported having more than 20 years of professional experience. Results showed that all participants used to give medical advice over the phone and 61.7% of them considered that such practices should be reimbursed. Among all participants, 76 (63.3%) were aware of the ban on telephone prescriptions that was implemented by the Lebanese order of physicians, and only 56 (73.7%) among them declared the reasons behind this restrained activity. The reported reasons were common medical practice behavior (49.1%), avoidance of useless medical visits (22.8%), parents' difficult economic status (12.3%), better communication (8.8%), and saving time (7.0%).

**Table 1:** Participants' clinical characteristics, phone consultations, and prescription-related variables.

	**N (%)**
Male, *n* = 120	90 (75.0)
**Pediatric specialty**, *n* = 120	
General pediatricians	77 (64.2)
Pediatric subspecialists	43 (35.8)
Neonatology	16 (37.2)
Pulmonology	7 (16.3)
Hematology and oncology	4 (9.3)
Cardiology	3 (7.0)
Endocrinology	3 (7.0)
Critical care	3 (7.0)
Infectious disease	3 (7.0)
Nephrology	2 (4.7)
Neurology	2 (4.7)
**Practice experience**, *n* = 120	
<10 years	51 (42.5)
10–20 years	36 (30.0)
>20 years	33 (27.5)
**Workplace**, *n* = 120^*^	
Private clinic	93 (77.5)
Teaching hospital	59 (49.2)
Private hospital	53 (44.2)
Dispensary	42 (35.0)
Governmental hospital	21 (17.5)
**Telephone consultations**	120 (100.0)
To consider telephone consultations as a reimbursable service	74 (61.7)
To be aware of the telephone prescriptions' restriction	76 (63.3)
Request for a family tie during a telephone consultation	109 (90.8)
Providing medical advice over the phone for first-time parents	49 (40.8)
Rate of medical follow-ups after a telephone consultation (for the same complaint)	
0–25%	37 (30.8)
25–50%	48 (40.0)
>50%	35 (29.2)
**Telephone prescriptions**, *n* = 120	109 (90.8)
**Frequency of daily telephone prescriptions**, *n* = 109	
<5 times	92 (84.4)
5–15 times	16 (14.7)
>15 times	1 (0.9)
**Communication tools**, *n* = 109^*^	
Direct call	88 (80.7)
SMS	30 (27.5)
WhatsApp application	67 (61.5)
Text message	59 (88.1)
Voice message	26 (38.8)
Voice call	21 (31.3)
Video call	4 (6.0)
**Verification of the prescribed drugs-related information and instructions**, *n* = 109	
During the same phone call	101 (92.7)
By sending an SMS after hanging up	75 (68.8)
**Pharmacist intervention after physician telephone prescription**, *n* = 109	44 (40.4)

* *More than one choice is applied*.

**Table 2:** Distribution of collected variables regarding clinical practices and telephone consultations between phone prescribers and non-prescribers.

	**Phone prescribers**		**F**	***p*-value**
	**No**	**Yes**		
	**N (%)**			
**Pediatric specialty**				
General pediatrician	6 (54.5)	71 (65.1)	0.488	0.521^a^
Pediatric subspecialist	5 (45.5)	38 (34.9)		
**Practice experience**				
<10 years	3 (27.3)	48 (44.0)	6.857	**0.020**^a^
10–20 years	1 (9.1)	35 (32.1)		
>20 years	7 (63.6)	26 (23.9)		
**Workplace**				
Private clinic	7 (63.6)	86 (78.9)	1.335	0.265^a^
Teaching hospital	3 (27.3)	56 (51.4)	2.232	0.205^b^
Private hospital	4 (36.4)	49 (45.0)	0.299	0.753^a^
Dispensary	4 (36.4)	38 (34.9)	0.010	1.000^a^
Governmental hospital	4 (36.4)	17 (15.6)	2.985	0.100^a^
To consider telephone consultations as a reimbursable service	3 (27.3)	71 (65.1)	6.060	**0.021**^a^
To be aware of the telephone prescription's restriction	11 (100.0)	65 (59.6)	7.011	**0.007**^a^
Providing medical advice over the phone for first time parents	2 (18.2)	47 (43.1)	2.572	0.196^a^
**Chief complaints during a telephone consultation**				
Fever	6 (54.5)	95 (87.2)	7.973	**0.015**^a^
Cough and rhinorrhoea	6 (54.5)	86 (78.9)	3.313	0.126^a^
Diarrhea	5 (45.5)	81 (74.3)	4.098	0.052^a^
Nausea or vomiting	4 (36.4)	67 (61.5)	2.606	0.121^a^
Skins issues	–	30 (27.5)	4.037	0.063^a^
Eyes problems	–	23 (21.1)	2.871	0.121^a^
Abdominal pain	–	8 (7.3)	0.865	1.000^a^
Otalgia	–	7 (6.4)	0.750	1.000^a^

a *Fisher exact test*.

b *Chi-square test*.

*Bold values are significant p-values*.

**Chart 1:**
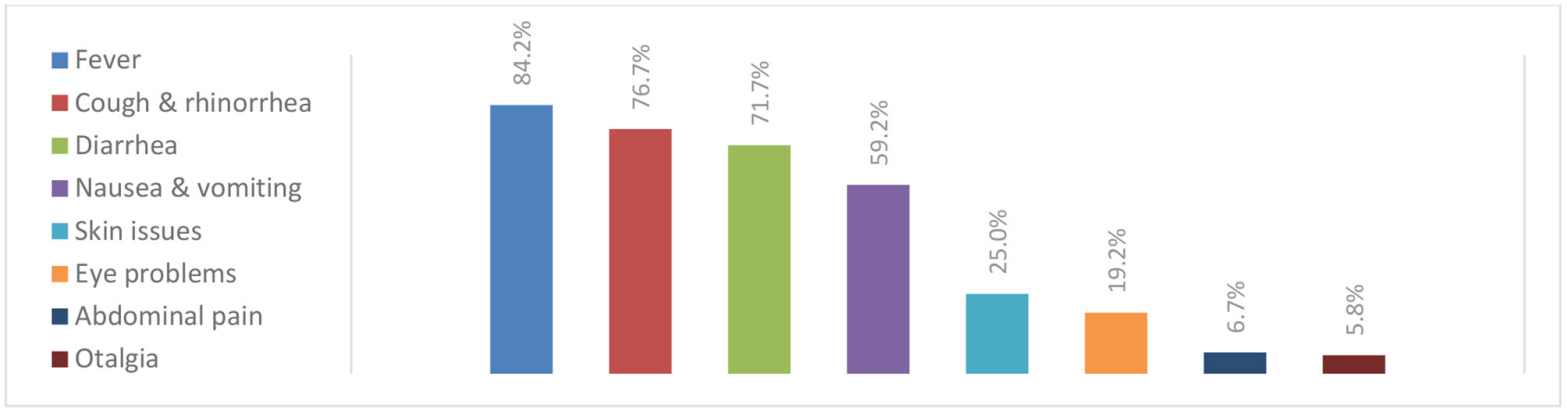
Chief complaints during a telephone consultation, *n* = 120.

**Chart 2:**
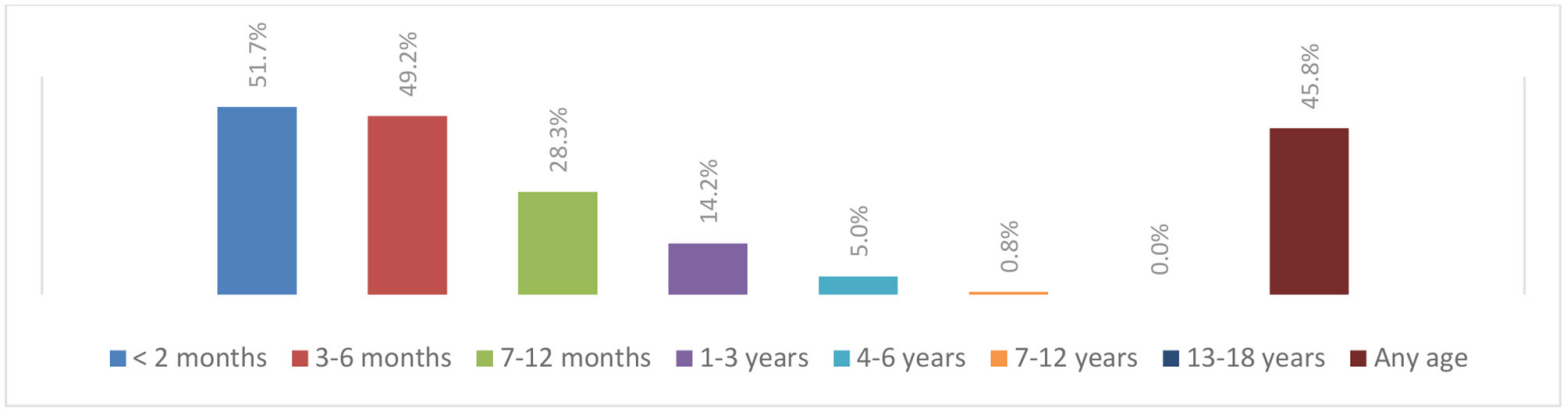
Patient's age from which pediatricians believe that a medical visit following a telephone consultation is mandatory, *n* = 120.

When participants were questioned about their behaviors related to telephonic medical consultations, 90.8% asked the caller about their family relationship with the child before giving any health-related advice while 40.8% gave healthcare information over the phone for first-time parents. In addition, 29.2% of the participants noted that, following a telephone consultation, more than 50% of their patients re-consulted them for the same complaint. The most frequent chief complaints reported during a telephone consultation were fever (84.2%), cough and rhinorrhea (76.7%), followed by diarrhea (71.7%), and vomiting (59.2%). Less common complaints were abdominal pain (6.7%) and ear pain (5.8%) (Chart 1). Besides, 51.7% of the participants considered that babies aged <2 months should always be examined after any phone consultation and this rate progressively decreased as the age of patients increased (Chart 2).

**Chart 3:**
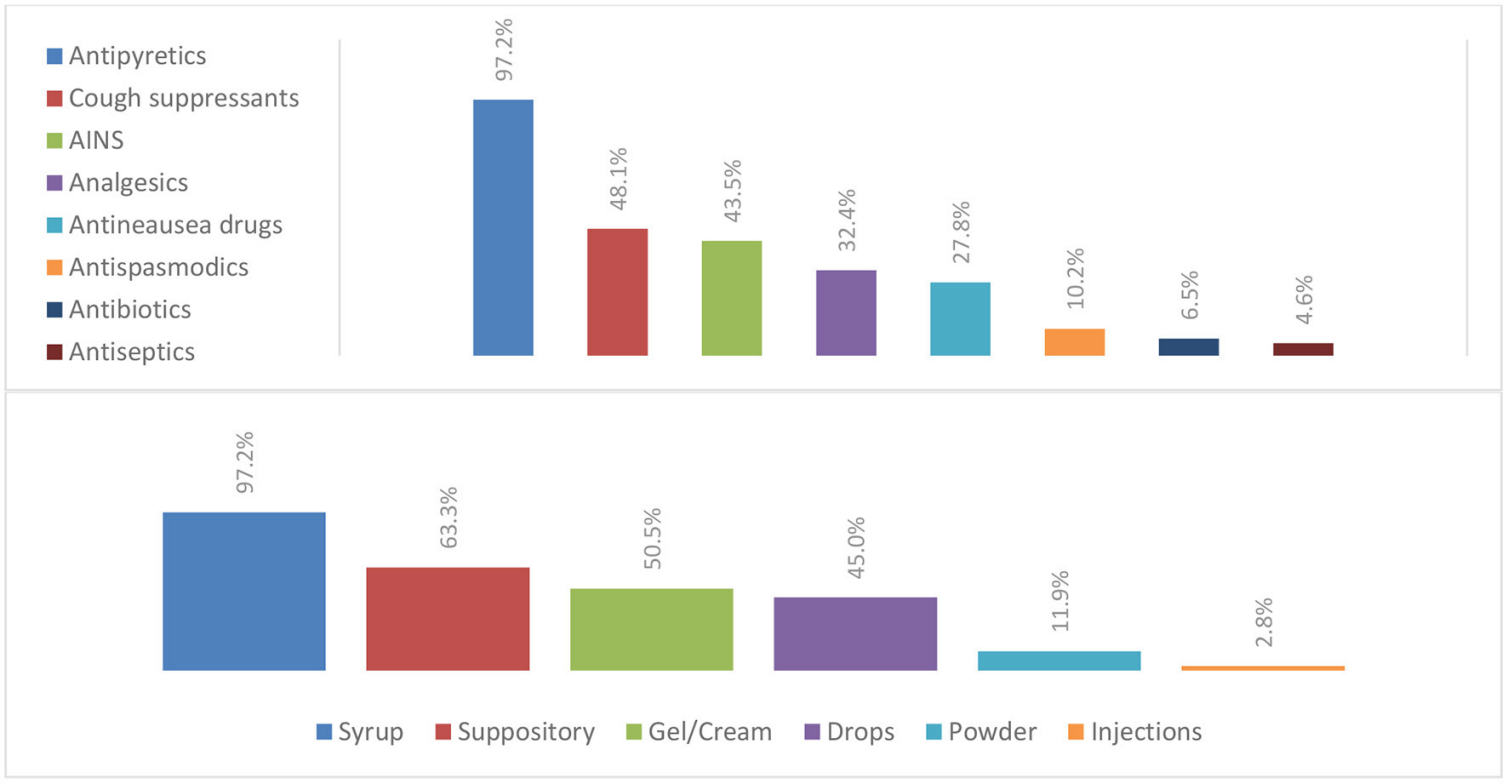
Phone-prescribed drugs and their different forms.

Among all participants, 90.8% prescribed medications over the phone, using direct calls in 80.7%, SMS in 27.5%, and WhatsApp application in 61.5% of cases. Most physicians made <5 prescriptions daily. The most often prescribed drugs over the phone were antipyretics (97.2%) followed by cough suppressants (48.1%) (Chart 3). Antibiotics prescriptions over the phone were attested in only 6.5% of the cases. Syrup and suppositories were the most common drug forms and only 2.8% of phone-prescribed drugs were in the form of injections (Chart 3).

**Chart 4:**
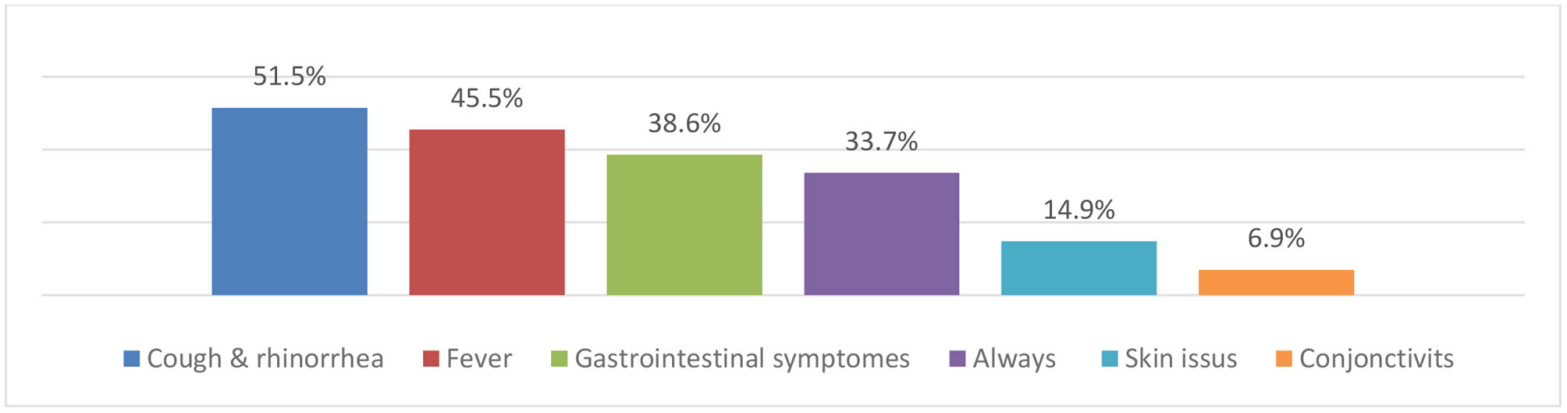
Chief complaints for which a follow-up is requested after a telephone prescription, *n* = 101.

When participants were asked about their behaviors following phone prescriptions, 33.7% stated that they always asked for a clinical follow-up independently of the type of medical complaint. Cough (51.5%) and fever (45.5%) were the most common complaints for which a medical visit was requested following a telephone prescription (Chart 4). Furthermore, prescribers used to verify the related information and instructions regarding drugs during the same phone call in 92.7% of the cases or by sending a text message after hanging up in 68.8% of cases. Results also showed that, in 40.4% of cases, physicians declared that pharmacists intervened to verify the accuracy of their telephonic prescriptions.

Distribution of the collected variables about clinical practices and telephone consultations between phone prescribers and non-prescribers is summarized in Table 2. Results showed that non-prescribers were more likely to have more than 20 years of experience when compared to prescribers (63.6 > 23.9%, *p* = 0.02). Furthermore, it seems that prescribers were more likely to affirm that telephone consultations should be reimbursed (65.1 > 27.3%, *p* = 0.021) and were less aware of the telephone prescription ban (59.6 <100%, *p* = 0.007). Finally, among all chief complaints reported during telephone consultations, fever was the only symptoms statistically associated with phone prescriptions.

## Discussion

Telephone consultation is defined as the use of telephone and wireless communication technology to deliver clinical healthcare information (6). Our study shows that telephone medical consultation is a very common practice among participating pediatric-trained physicians. When compared to a face-to-face consultation, this practice seems to be suitable for patients' parents since it allows them to access rapid and direct healthcare information with higher cost-effectiveness (8–10). Lin et al. even reported that almost half of the surveyed patients in ambulatory internal medicine clinics are ready to pay a median amount of two dollars per message for online correspondences with their physicians (11). Another study performed by Kleiner et al. stated that the majority of surveyed parents in pediatric clinics have shown an interest in electronic communication with their physician (12). Both patients and physicians consider that providing healthcare information over the phone reduces waiting times for health services, increases patient satisfaction (13), and improves health outcomes and processes of care (3). These facts could also explain the high rate of telephone prescriptions among participants, with a significant majority thinking that they should be reimbursed for this service. It seems that physicians feel compelled to interact with their patients over the phone even though they have been advised by concerned authorities to avoid this type of practice. This may lead to an increased daily burden, decreased safety of practice, and subsequent request of reimbursement with such a high prevalence. This prevalent finding could be also explained by the fact that anxious parents, confronted with stressful situations, are relieved once they consult their child's pediatrician. It was highlighted by Riva et al., who reported that cell phones were shown to be effective in reducing the stress level in real-life situations with a significant decrease in anxiety scores (14).

On the other side, 9.1% of participants reported that they did not prescribe drugs and medication over the phone. Phone-based healthcare consultations should complement the clinical pathway (history of the patient, examination, and the treatment prescription) rather than replacing it. It has been reported that healthcare providers may misinterpret health information delivered over the phone, as an endpoint to their responsibilities within care delivery, thinking that their work is completed once the message is delivered. This can lead to an inappropriate follow-up of patients and may not immediately meet patients' needs. Gurol-Urganci et al. reported in their review that there is currently insufficient evidence regarding the benefits and risks associated with phone messaging to communicate medical investigations' results, and that patients' safety may also be compromised if the prescription information is not applied appropriately (13). Kleiner et al. also reported that most of the pediatricians are against online electronic communication with their patients, driven by the fear of an extra workload and inappropriate messaging, citing their high concerns for a potential increase in medical liability and risks for patients' privacy and confidentiality (12). Foster et al. also attested low levels of confidence among British clinicians providing telephone-based care and highlighted the potential risks of missing a serious condition (15). Physicians may also fear that the adoption of this simple way of communication will encourage patients to send unnecessary messages resulting in an increased flood of inquiries.

In our study, participants reported that the most common means of communication used by parents to consult them were phone calls and text messaging. The mobile phone is one of the most important and inexpensive means of communication used worldwide, offering an instant and low-cost transmission of health-related information, as confirmed by several studies (16). It has also been demonstrated that mobile phones can improve the processes of access and use of healthcare services, enhance the efficiency of service delivery, improve diagnosis and treatment, and, finally, support public health programs. Mobile phone messaging has been also used to communicate results of medical investigations (13), to provide reminders for attendance at healthcare appointments (17), to improve patient understanding and compliance with medications, to facilitate self-management of long-term illnesses (18), to monitor chronic conditions (19, 20), and to provide psychological support (21). The risk of misinterpretation of electronically sent messages exists, but it is weak since patients can ask for immediate clarification and can accurately store contents with a high level of confidentiality and privacy (13). Houston et al. showed that patients also appreciate the possibility of being able to save and re-read medical advice or other important information that is backed up on their mobile devices (22).

According to the American Academy of Pediatrics, fever is one of the common chief clinical complaints managed by pediatricians that lead to unscheduled physician visits and urgent telephone consultations for advice on its control and the use of different over-the-counters antipyretics (23). These facts were highlighted by our findings, and we believe that antipyretic therapy is commonly used by parents and generally encouraged by pediatricians (24). Parents consider healthcare providers as the primary source of information on fever management and consider them responsible for providing appropriate counseling about fever and the use of antipyretics. Parents are also highly concerned with the need to achieve normothermia and may administer antipyretics even though there is minimal fever to avoid associated morbidities (25).

Our results also showed a low prescription rate of antibiotics over the phone. Despite this promising practice, earlier studies conducted in Lebanon reported a high prevalence of inappropriate practice among community pharmacists in dispensing antibiotics without a prescription and among parents when administering them to their child (26–28). For that, we believe that educational campaigns are warranted to increase awareness on antibiotics misuse in pediatrics, and severe legislative actions should be taken to restrict such practices in order to prevent poor clinical outcomes. In this regard, Marc et al. reported that French general practitioners in pediatrics primary care face difficulties of identifying bacterial infections and limiting antibiotics' use. Most importantly, they also reported that lack of time during consultations is one of the main reasons behind inappropriate antibiotics prescriptions (29).

Surprisingly, we reported that 9.2% of our participants did not request a family tie before giving any health-related pieces of advice, and 40.8% had no problem with giving healthcare information over the phone for first-time parents. Car et al. reported that, in order to ensure appropriate quality of telephone consultations, a standardized documentation approach is needed. Further, the caller's name and all their related information should be obtained, and all calls should be recorded, following patients' consent (6). On the other hand, we believe that during a telephone consultation, the safety of the given information is significantly met as our participants closely monitor and evaluate prescribed drugs-related information and instructions by summarizing the main points covered or by texting back information after hanging up (6). We also believe that good organization of telephone-based health management helps the physician to pay appropriate attention to their caller, and this could be established by a healthcare assistant who initially triages calls and can also provide the option of a call-back appointment. Moreover, Wallwiener et al. reported that secure electronic messaging between patients and physicians is a useful, convenient, and time-saving addition to the routine healthcare phone-based infrastructure (30). Identification of secure providers and integration of physician reimbursement systems and messaging into medical records are considered to be promising improvements that will speed up the adoption of this practice by larger healthcare organizations.

Finally, Beal stated that pediatric medication errors are a common and serious problem in the United States, and they can be effectively controlled by implementing educational and counseling strategies (31). Gilligan et al. reported that the most common reasons for pharmacists' intervention on e-prescriptions were excessive quantity/duration and violation of legal requirements, and that intervention rates did not change when compared to handwritten prescriptions (32). Carroll NV reviewed the influence of community pharmacists in the United States on drug prescribing and reported that pharmacists routinely intervene in traditional practices and have a significant impact on clinicians' prescriptions (33). Through this statement, our results showed that verification and correction of telephone prescriptions by pharmacists have been noted in 40.4% of cases, highlighting the high rates of avoidable medication errors. Nevertheless, Brown et al. declared in their study that community pharmacists did not appropriately and consistently identify drug prescription errors or implement interventions known to decrease the likelihood of these incorrect prescriptions (34). For that, we believe that phone-based medical activities can put physicians at serious and unavoidable risk of malpractice and may endanger children's health, especially when more than half of the participants reported that their patients frequently revisited them for the same complaint even after a phone consultation. As these activities seem to be highly prevalent, pharmacists should intervene correctly to limit drug prescription errors only when trained enough to provide a patient-centered style of pharmaceutical practice (33).

## Study Limitations and Strengths

The present study is one of the first in our country to focus on the emerging area of telehealth in the form of phone-based consultations and prescriptions. We did not obtain the corresponding data on whether the child had been examined by the pediatrician in the 24–48 h before giving phone advice and prescription. This information could help in establishing future clinical practice guidelines on phone consultations. Medicine and healthcare systems will surely develop in this area, using technology as a way to provide better support, and there is thus significant interest in the exploitation of this research topic. We believe that the study population was well-chosen since pediatricians are considered to be among the most exposed physicians to this type of practice. Our study is based on one medical specialty, and, thus, presents the overall impact of these phone-based practices. Our results could lead to future research topics, focusing on specific issues of telephone consultations and the real clinical impact of phone prescriptions in all healthcare fields.

## Conclusion

Effective communication between patients' parents and pediatricians involves the accurate and timely transmission of information while maintaining confidentiality and using strategies to minimize misunderstanding or misinterpretation of information. Our study reveals that telephone consultations between patients' parents and their doctors are a highly prevalent means of communication in pediatric practices, despite restrictions, highlighting their actual utility and necessity while pointing out the increased non-reimbursed daily workload. Prescribing drugs over the phone can surely be a relevant advantage but can sometimes lead to serious health-related risks. Children are vulnerable subjects with unique characteristics and it is therefore mandatory to develop an efficient, secure, and confidential electronic infrastructure that respects the rights and duties of doctors as well as patients. We believe that telehealth holds potential in our country. However, there is a relevant need for clinical practice guidelines and a regulatory framework developed by pediatricians along with the pharmacists to ensure the safety of both patients and healthcare providers.

## Data Availability Statement

The raw data supporting the conclusions of this article will be made available by the authors, without undue reservation, to any qualified researcher.

## Ethics Statement

The studies involving human participants were reviewed and approved by Saint Joseph University Research Ethics Committee. The patients/participants provided their written informed consent to participate in this study.

## Author Contributions

RH and CS took the lead in writing the manuscript. RH double-checked collected data, performed statistical calculations, analyzed, and critically interpreted the results. BE conceived the presented idea and supervised the project. All authors discussed the results and read and approved the final manuscript.

### Authorship Declaration

All authors listed meet the authorship criteria according to the latest guidelines of the international committee of medical journal editors, and all authors are in agreement with the manuscript.

### Conflict of Interest

The authors declare that the research was conducted in the absence of any commercial or financial relationships that could be construed as a potential conflict of interest.
